# Research progress on the neural circuits mechanisms of anxiety

**DOI:** 10.3389/fncir.2025.1609145

**Published:** 2025-06-25

**Authors:** Wenxuan Gong

**Affiliations:** School of Life Science, Beijing University of Chinese Medicine, Beijing, China

**Keywords:** anxiety, neural circuit, bed nucleus of the stria terminalis, amygdala, lateral habenula

## Abstract

Anxiety disorders, as a critical mental health issue, profoundly impact an individual’s quality of life and social participation while imposing a considerable economic burden on communities. This underlines the urgent need for in-depth studies on the mechanisms underlying anxiety-like behaviors. These mechanisms are overseen by intricate neural regulatory networks, and the understanding of them has significantly advanced in recent decades, largely due to breakthroughs in neuroscience. Traditionally, research on brain regions controlling anxiety responses has been focused on key brain regions. However, recent studies have expanded this scope to encompass a broader network, including the amygdala, the bed nucleus of the stria terminalis (BNST), and the lateral habenula (LHb). Each of these regions plays a distinct role in mediating specific components of anxiety-like behaviors: the amygdala is central to emotional processing, the BNST contributes to the prolonged state of anxiety, and the LHb is pivotal in encoding negative signals that amplify aversive emotions. This review underscores the evolving and interconnected nature of these neural circuits, illustrating the intricate interplay in shaping anxiety-like behaviors. By proposing a layered representation of the neural circuitry, this study aims to unravel the neurobiological basis of anxiety-like behaviors, paving the way for more effective therapeutic strategies. These insights hold promise for advancing treatment approaches that could alleviate the burden of anxiety disorders in the future.

## 1 Introduction

Anxiety disorders rank among the most prevalent psychiatric conditions worldwide, typically emerging in childhood and affecting over 25% of individuals across their lifespan (Balogh et al., 2021; Kessler et al., 2005). The substantial personal and societal burden associated with these conditions underscores the urgent need to elucidate their neurobiological underpinnings (Craske et al., 2009). In this context, preclinical research employing murine models has become indispensable for investigating anxiety-related behavioral phenotypes and their neural correlates. Mice exhibit evolutionarily conserved anxiety-like behaviors in response to environmental challenges, paralleling key features of human anxiety responses (LeDoux and Pine, 2016). Notably, cross-species genetic homology has been identified in critical pathways - such as polymorphisms in the brain-derived neurotrophic factor (BDNF) gene - that modulate anxiety-like behavioral profiles under stress in both mice and humans (Chen et al., 2006; Montag et al., 2010). These behavioral manifestations can be systematically quantified through standardized paradigms including elevated plus maze, open field tests, which serve as translational bridges to human anxiety mechanisms (Schoenfeld et al., 2014). Furthermore, recent technological advancements have greatly accelerated the research on the neurobiological mechanisms underlying anxiety disorders. Cutting-edge approaches such as chemogenetics, optogenetics, immunohistochemistry, fiber photometry, and electrophysiological methods have facilitated in-depth exploration of anxiety-related brain regions, providing novel insights and potential therapeutic strategies (Balogh et al., 2021; Swanson et al., 2022). Moreover, fear and anxiety, though often intertwined, differ fundamentally: fear is a rapid, reflexive response to a specific, immediate threat (e.g., freezing in the presence of a predator), while anxiety is a more sustained, anticipatory state triggered by vague or future-oriented threats (e.g., chronic worry) (Daniel-Watanabe and Fletcher, 2022). This distinction is critical for understanding the neural circuits of anxiety-like behaviors.

In recent years, significant advancements have been made in understanding the neural mechanisms of regulating anxiety. Researchers have identified several key brain regions involved in anxiety processing, including the bed nucleus of the stria terminalis (BNST), amygdala, lateral habenula (LHb), hippocampus (HPC) and prefrontal cortex (PFC), and so on. Studies consistently demonstrate hyperactivation of the amygdala across various anxiety disorders both in rodent and in human (Tye et al., 2011; Amit Etkin and Tor, 2007). The amygdala is a critical structure for emotional processing. The basolateral amygdala to the central amygdala (BLA-CeA) pathway plays a crucial role in emotional memory and exhibit anxiolytic effects (Tye et al., 2011). Additionally, the amygdala forms extensive connections with BNST, ventral hippocampus (vHPC), and medial prefrontal cortex (mPFC), establishing essential neural circuits that underlie the expression of anxiety-like behaviors and emotional memory (Poulos et al., 2010; Felix-Ortiz and Tye, 2014; Tejeda and O’Donnell, 2014). Notably, studies indicate that activation of the BLA-vHPC pathway increases anxiety-like behaviors, whereas its inhibition exerts anxiolytic effects (Felix-Ortiz et al., 2013). The BNST is another key region implicated in anxiety disorders, with its subregions, including its subregions-the oval nucleus (ov) of the BNST (ovBNST), the anterodorsal of the BNST (adBNST) and the ventrolateral BNST (vlBNST), also plays a central role in the development of anxiety disorders (Kim et al., 2013; Jennings et al., 2013). Notably, the ovBNST and adBNST exhibit opposing roles in modulating anxiety, influenced by both their interaction and specific neural pathways (Kim et al., 2013). Previous optogenetic studies revealed that inhibition of the ovBNST, produced anxiolytic effects in rats, while reducing BLA fiber terminal activity in the adBNST increased anxiety-like behaviors (Kim et al., 2013). Furthermore, BLA inputs to the adBNST and to vlBNST project to the ventral tegmental area (VTA), releasing multiple neurotransmitters that further influence anxiety regulation (Kim et al., 2013; Jennings et al., 2013). Additionally, the HPC, particularly its subregions such as the dentate gyrus (DG) and CA3, plays a crucial role in anxiety-related behaviors (Engin et al., 2016). The synchronized activity between the HPC and PFC is a key factor in modulating anxiety responses (Felix-Ortiz et al., 2016; Parfitt et al., 2017; Likhtik et al., 2014). In addition, the LHb has also emerged as a critical role in modulating responses to anxiogenic stimulus. Located between the dorsomedial thalamus and the third ventricle (Hikosaka, 2010), reductions in LHb volume and neuronal density have been linked to increased anxiety-like behaviors (Jacinto et al., 2017). The LHb sends glutamatergic projections to the rostromedial tegmental nucleus (RMTg), which inhibits midbrain DA cell firing via GABAergic innervation of the VTA (Jhou et al., 2009). Moreover, noradrenergic (NE) system, through locus coeruleus (LC) projections to the LHb has been implicated in the neural circuits underlying heightened anxiety responses (Purvis et al., 2018).

In this review, we specifically focus on anxiety-like behaviors, synthesizing recent advancements in neuroscience to explore the neural circuits underlying anxiety disorders. By examining the role of key brain regions, including the BNST, amygdala, LHb, as well as other regions involved anxiety, such as HPC and PFC, aiming to further elucidate precise pathophysiology of anxiety-like behaviors, enhance the effectiveness of individualized treatment approaches, and pave the way for the development of innovative therapeutic strategies.

## 2 The role of BNST in anxiety regulation

**Figure 1:**
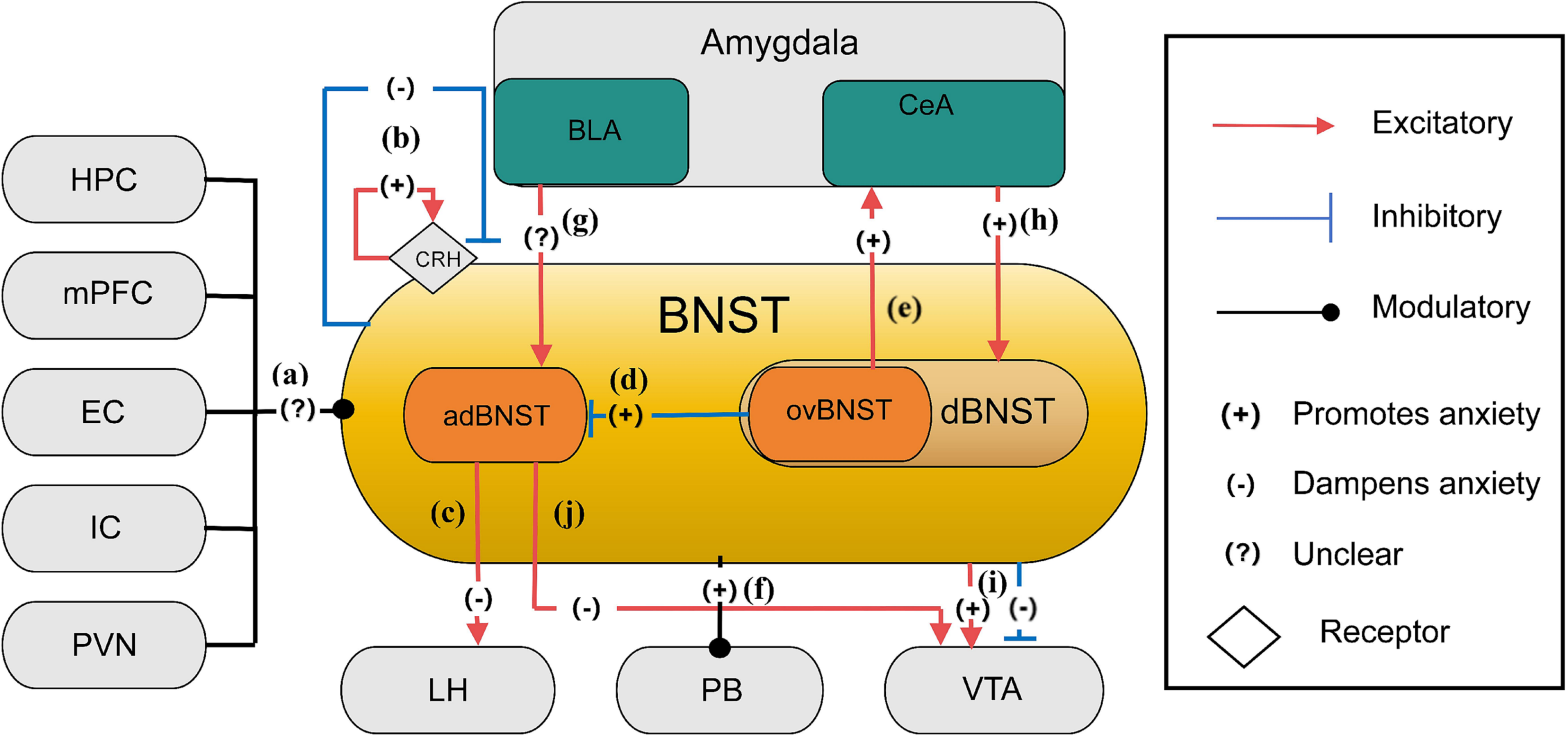
The anxiety network of BNST (Ahrens et al., 2018; Calhoon and Tye, 2015; Crowley et al., 2016; Figel et al., 2019; Jennings et al., 2013; Kim et al., 2013). **(a)** Inputs to the BNST come from various brain regions, including the hippocampus, mPFC, entorhinal cortex, insular cortex, and PVN, cumulatively shaping anxiety control through their collective influence; **(b)** CRH receptor stimulation and lesioning within the BNST regulate anxiety levels, leading to specific localized impacts that either sustain or diminish anxiety; **(c)** Neurons from the adBNST to the lateral hypothalamus (LH) likely play key roles in reducing anxiety behaviors. The adBNST-LH pathway subtly adjusts anxiety-related behavior; **(d)** In the BNST, GABAergic neurons, especially those located in the ovBNST, have an inhibitory effect over the adBNST, leading to a relatively increase in anxiety throughout this neural network; **(e)** The ovBNST-CeA pathway is an excitatory connection which promotes anxiety. This neural pathway, connecting ovBNST to CeA, plays an essential role in the modulation of anxiety-related behaviors. Activation of ovBNST-CeA enhances anxiety states; **(f)** Stimulation of the BNST-PB projection intensifies anxiety levels in stressful settings**; (g)** Enhancing glutamatergic BLA projections elevates adBNST neuron function, emphasizing the intricate excitation modulation of anxiety by the BNST; **(h)** The excitatory pathway from the amygdala’s central nucleus to the BNST increases anxiety by elevating dBNST neuron activity through reducing inhibitory signals; **(i)** The BNST projects to the VTA eliciting mixed responses, including glutamatergic neurons and GABAergic neurons, which may lead to excitatory increased anxiety and inhibitory reduced anxiety, illustrating an BNST-VTA pathway in the modulation of anxiety; **(j)** The adBNST extends its influence to the VTA and engages with key brain regions associated with anxiety, such as the LH and PB, thereby diminishing anxiety via this network.

Recent studies have significantly advanced our understanding of anxiety-related neural pathways, particularly by examining specific neuron types within the BNST subregions of murine models. The BNST, a small yet critical brain structure, plays a pivotal role in mediating sustained anxiety states and behavioral responses to ambiguous threat (Davis et al., 2010). As a key node in the extended amygdala macrostructure, the BNST integrates information from limbic regions to orchestrate prolonged anxiety states distinct from acute fear processing in the central amygdala (Ueda et al., 2021). Located in the basomedial forebrain, it is positioned near the globus pallidus, fornix, internal capsule, and lateral ventricle, extending from the anterior commissure to the hypothalamus (Clauss et al., 2019; Avery et al., 2014). As a central hub integrating information from brain regions processing threat, context, and stress, the BNST receives inputs from regions such as the HPC, amygdala, medial prefrontal cortex (mPFC), entorhinal cortex, insular cortex, and paraventricular nucleus of the hypothalamus (PVN) (Calhoon and Tye, 2015; Arakawa et al., 2023; McDonald, 1998; Christianson et al., 2011) (Figure 1a). It then projects outputs to areas such as the VTA, lateral hypothalamus (LH), and parabrachial nucleus (PB), all of which orchestrate specific behavioral (e.g., avoidance, risk assessment) and physiological (e.g., HPA axis activation, autonomic changes) responses characteristic of anxiety (Kim et al., 2013; Adhikari, 2014).

The BNST functions as a neural switchboard, integrating and modulating stress-related information across multiple pathways (Steimer, 2002; López et al., 1999). This role is further underscored by its interaction with the hypothalamic–pituitary–adrenal (HPA) axis, facilitating the release of stress hormones to prepare the body for potential threats (Fox et al., 2015). Within the extended amygdala paradigm, the BNST and central amygdala constitute a continuous macrostructure where the BNST specializes in processing unpredictable threat contexts, contrasting with amygdala nuclei handling discrete threats (Urien and Bauer, 2021). In this complex neuroanatomical framework, the inhibitory GABAergic system and excitatory glutamatergic system play crucial roles in modulating neuronal excitability within BNST circuits that drive anxiety-like behaviors (Kim and Kim, 2021; Li et al., 2012). Specifically, in the ovBNST, GABAergic neurons exert strong inhibitory effects on the adBNST, contributing to heightened anxiety responses (Kim et al., 2013; Tovote et al., 2015). The release of *γ*-aminobutyric acid (GABA) suppresses excitatory signals, reducing neural responsiveness to stressors. Conversely, glutamatergic neurons within the adBNST mediate anxiety-reducing effects, counterbalancing the inhibitory activity of the ovBNST. Studies indicate that reducing glutamatergic signaling via mGlUR agonists can mitigate HPA axis overactivation, producing anxiolytic effects (Scaccianoce et al., 2003; Muly et al., 2007). Hence, targeting mGLURs in the BNST presents a promising strategy for anxiety disorders. Additionally, BNST neurons co-express GABA and dynorphin, functioning as a modulatory switch that fine-tunes glutamatergic activity, a mechanism also observed in the HPC and hypothalamus (Crowley et al., 2016; Belzung et al., 2006). Optogenetic activation of these GABA/dynorphin neurons induces dynorphin release, which binds presynaptic *κ*-opioid receptors (KORs) on glutamatergic terminals to suppress neurotransmitter release probability (glutamate inhibition) (Crowley et al., 2016). Conversely, KOR activation independently suppresses GABA release via ERK-dependent presynaptic mechanisms (GABA inhibition) (Li et al., 2012). The intricate interplay among GABA, glutamate, and dynorphin within the BNST creates a complex neural architecture orchestrating behavioral outputs to stress (Crowley et al., 2016). Critically, glutamatergic modulation exhibits pathway specificity (preferentially inhibiting BLA inputs) and cell-type selectivity (stronger suppression on dynorphin-positive neurons) (Crowley et al., 2016), while GABAergic inhibition gates long-range inputs like the CeA-BNST pathway (Li et al., 2012). By selectively targeting these distinct neuromodulatory systems, novel pharmacological interventions could be developed to regulate this neural network and mitigate anxiety-like behaviors (Li et al., 2012; Crowley et al., 2016). Besides, the crucial role of the BNST in sustained anxiety states is underscored by the involvement of corticotrophin-releasing hormone receptors, whose activation is essential for sustaining anxiety states (Steimer, 2002). Notably, studies have demonstrated that lesions in specific BNST regions exacerbate anxiety, whereas optogenetic activation of certain BNST subregions alleviates anxiety, underscoring the potential for precise, circuit-specific therapeutic strategies (Kim et al., 2013; Figure 1b). Finally, recent studies have significantly advanced the understanding of anxiety-related neural pathways, primarily by examining specific neuron types within BNST subregions in mice. These findings shed light on the complex neural mechanisms underlying anxiety regulation. Within anterolateral BNST (alBNST) of mice, NPY-expressing GABAergic interneurons play a crucial role in reducing anxiety-like behaviors, potentially by enhancing inhibitory tone. These neurons influence GABAergic signaling, resembling hyperpolarizing Type II neurons in the BNSTALG, thereby contributing to anxiety reduction and highlighting the intricate nature of the surrounding neural network (Van de Poll et al., 2023). In the ovBNST, Type III neurons are of particular interest. Among the 10% of BNSTALG neurons that express CRF, which are primarily GABAergic and glutamatergic, projections extend beyond the BNST to regions linked to anxiety regulation. These projections influence neurotransmitter release and neural activity, deepening the understanding of BNST-mediated anxiety mechanisms (Van de Poll et al., 2023). CRF-expressing neurons in the ovBNST play a significant role in anxiety modulation. Their GABAergic and glutamatergic composition and distinct projection patterns significantly influence neural circuit functions related to anxiety, particularly within key regions such as NAc, VTA and CeA, which are integral to anxiety control (Van de Poll et al., 2023). Another key neuronal population in the ovBNST consists of SOM-neurons, which exhibit distinctive characteristics. The distribution of BNST projections, their lack of co-expression with PKCδ-positive cells, and their role in forming long-range projections to the periaqueductal gray (PAG) provide novel insights into the function of these projections in anxiety-related defensive behaviors. GABAergic SOM-neurons projecting positively from the BNST to the NAc interneurons regulate anxiety-like responses, refining the understanding of BNST-driven anxiety circuits (Van de Poll et al., 2023). Additionally, studies on PKCδ-expressing neurons in the ovBNST have revealed that their activation is context-dependent, influenced by factors such as stress and sex, which in turn alter behaviors such as risk assessment (e.g., stretch-attend postures) and avoidance in anxiety tests. This research has deepened our comprehension of the BNST’s role in anxiety processing, paving the way for targeted therapeutic strategies (Van de Poll et al., 2023).

Specifically, activation of neuronal projections from the adBNST to the lateral hypothalamus (LH) reduces anxiety-like behaviors, suggesting its significant role in anxiety mitigation (Ahrens et al., 2018). Current hypotheses suggest that neuronal projections from the adBNST to the lateral hypothalamus (LH) play a central role in mitigating anxiety-related behaviors (Kim et al., 2013). The adBNST-LH pathway subtly influences anxiety alleviation by modulating behavioral states, such as risk-avoidance behavior, while exerting minimal effects on physiological or hunger responses. This highlights the adBNST’s central function in orchestrating specific aspects of anxiety-related behavioral output and its potential as a therapeutic target for anxiety disorders (Kim et al., 2013; Figure 1c). Conversely, the ovBNST is linked to anxiogenic processes, actively intensifying anxiety by either inhibiting adBNST activity or through direct connections with anxiety-related structures such as the central amygdala (CeA) (Kim et al., 2013; Figures 1d,e). GABAergic neurons in the ovBNST, located within the stria terminalis’ dorsal bed nucleus (dBNST) are crucial in this regulatory process, indicating that ovBNST and adBNST exert opposing effects on anxiety regulation, with the ovBNST potentially exerting a greater impact on anxiety (Kim et al., 2013). The BNST’s interaction with the parabrachial nucleus (PB) further highlights its role in anxiety modulation. To be specific, BNST-PB projections regulate respiratory patterns in mice, reducing respiratory rates in contrast to the hyperventilatory response typically observed in fear. However, activation of the BNST-PB pathway in anxiety-inducing environments has been shown to exacerbate anxiety (Kim et al., 2013; Figure 1f). Additionally, the adBNST’s connectivity provides further insights into its role in anxiety. Stimulating glutamatergic projections from the BLA enhances adBNST neuronal activity, as demonstrated in Elevated Plus Maze Tests (EPMT), highlighting the delicate interplay between excitatory and inhibitory processes within the BNST (Kim et al., 2013; Calhoon and Tye, 2015; Figure 1g). The amygdala-BNST pathway is another key player in anxiety regulation. Research on mutant mice indicates that increased stimulation of SOM + neurons in the central nucleus of the amygdala (CeA) leads to elevated anxiety, as evidenced by EPMT and Open Field Test (OFT) outcomes. This increase in anxiety is linked to reduced inhibitory signaling, which results in heightened BNST neuronal activity (Ahrens et al., 2018; Figure 1h). Moreover, Kappa Opioid Receptors (KORs) in the BNST regulate anxiety by selectively suppressing neurotransmitter release from the BLA. The interaction between KOR-driven modulation and the activity of dynorphin (DYN)-positive neurons within the BNST, which regulate glutamatergic pathways, highlights a complex, cell-type-specific process. Notably, systemic activation of KORs has been found to block the anxiolytic effects mediated by the BLA-BNST circuit in the OFT, further emphasizing their role in anxiety regulation (Crowley et al., 2016). Clinical research indicates that individuals with social anxiety disorder (SAD) exhibit more stronger phasic reactions in the amygdala and BNST upon exposure to threat-related cues, compared to healthy controls (HC). This suggests that the BNST plays a critical role in the rapid regulation of anxiety, with immediate activation in response to anticipated threats contributing to heightened neural responses in anxiety disorders (Clauss et al., 2019; Figel et al., 2019). Further studies on the BNST’s projections to VTA neurons have revealed distinct anxiety-related stimuli. Glutamatergic neurons within the BNST-VTA pathway exhibit increased firing rates, while GABAergic neurons show decreased activity, reinforcing the functional dichotomy within this circuit (Geisler and Zahm, 2005; Jennings et al., 2013; Figure 1i). Experimental activation of BNST glutamatergic pathways in Vglut2-ires-cre and Vgat-ires-cre mouse models has been shown to induce anxiety-like and aversive behaviors. In contrast, stimulating GABAergic projections from the BNST to the VTA suppresses VTA GABAergic neurons, leading to reward-related behaviors and reduced anxiety, underscoring the BNST-VTA circuit’s dual role in anxiety modulation (Jennings et al., 2013). Given that the adBNST projects onto the VTA, its ability to reduce anxiety seems to be facilitated through its interaction with key brain regions associated with anxiety, such as the LH, PB, and VTA (Kim et al., 2013; Figure 1j). These findings collectively emphasize the BNST’s intricate role in anxiety regulation, offering promising avenues for targeted therapeutic interventions.

HPC (Hippocampus), mPFC (medial Prefrontal Cortex), EC (Entorhinal Cortex), IC (Insular Cortex), PVN (Paraventricular Nucleus of the Hypothalamus), BLA (Basolateral Amygdala), CeA (Central Amygdala), CeL (Central nucleus of the Amygdala lateral division), adBNST (anterodorsal nucleus of the Bed Nucleus of the Stria Terminalis), ovBNST (oval nucleus of the Bed Nucleus of the Stria Terminalis), dBNST (dorsal Bed Nucleus of the Stria Terminalis), LH (the Lateral Hypothalamus), PB (the Parabrachial Nucleus), VTA (the Ventral Tegmental Area), CRH (the Corticotrophin-releasing Hormone Receptors).

## 3 The role of the amygdala in anxiety regulation

The amygdala, a key neural structure, plays a crucial role in processing emotions, particularly anxiety (Forster et al., 2012). Situated within the temporal lobe, it is medially bordered by the pial surface above the semiannular gyrus, while superiorly, it is delineated by an imaginary line extending from the quadrigeminal cistern to the inferior circular sulcus of the insula (ÇİFTCİGG, 2021; Nikolenko et al., 2020). Structurally, the amygdala consists of multiple subdivisions, among which the BLA and CeA are particularly critical for orchestrating different aspects of anxiety-like behaviors (Janak and Tye, 2015). Functionally dissociable roles exist within its subnuclei: the BLA encodes conditioned threat associations (learned fear responses to specific cues) (Koo et al., 2004), while the CeA executes both phasic fear responses (e.g., acute freezing) and acute anxiety initiation (Ressler, 2010). Remarkably, these two regions originate from different cellular lineages and exhibit marked differences in their inhibitory neuron populations. The BLA, resembling cortical structures, primarily comprises excitatory principal projection neurons interspersed with a smaller population of local inhibitory interneurons. In contrast, the CeA shares more similarities with striatal regions, being predominantly composed of inhibitory neurons (Babaev et al., 2018; Gilpin et al., 2015). In the context of anxiety-related neural circuits, the amygdala receives afferent inputs from various brain regions, including the HPC, Prefrontal Cortex (PFC), and locus ceruleus (LC) (Felix-Ortiz and Tye, 2014; Tejeda and O'Donnell, 2014; Steimer, 2002), and projects to several areas such as the BNST, vHPC, anterior cingulate cortex (ACC), hypothalamus, brainstem, spinal cord, and dorsal vagal complex (DVC) (Felix-Ortiz et al., 2016; Calhoon and Tye, 2015; Babaev et al., 2018; Albrechet-Souza et al., 2009; LeDoux et al., 1988). These interconnected networks, centered on the amygdala, are essential for generating appropriate behavioral and physiological responses to potential threat, which are dysregulated in pathological anxiety. Dysfunction in these neural connections is believed to underlie the pathophysiology of anxiety-related behaviors, contributing to symptoms such as excessive worry, hypervigilance, and avoidance, which are hallmark features of anxiety disorders (Babaev et al., 2018). Understanding these complex amygdala-centered circuits provides critical insights into potential therapeutic targets for anxiety-related conditions.

The CeA is a core component of the ‘extended amygdala’ framework. This concept describes a macrostructure encompassing the central amygdala (CeA), the bed nucleus of the stria terminalis (BNST), and the intervening sublenticular region, forming a continuous anatomical and functional continuum along the rostrocaudal axis (Cassell et al., 1999). While sharing cytoarchitectural similarities, connectivity patterns, and neurochemical properties with the CeA, the BNST is distinguished by its role in processing sustained, context-dependent threats rather than acute, phasic stimuli (Brinkmann et al., 2018). Within this extended amygdala framework, the BNST is particularly critical for mediating sustained anxiety responses to diffuse, unpredictable, or long-duration threats, whereas the CeA primarily orchestrates immediate phasic fear reactions to discrete, acute danger cues (Brinkmann et al., 2018). This functional dissociation within the extended amygdala underscores the critical distinction between the neural substrates for conditioned fear (e.g., cue-specific responses mediated by CeA circuits) versus unconditioned anxiety (e.g., contextual apprehension sustained by BNST circuits) (Ueda et al., 2021).

**Figure 2:**
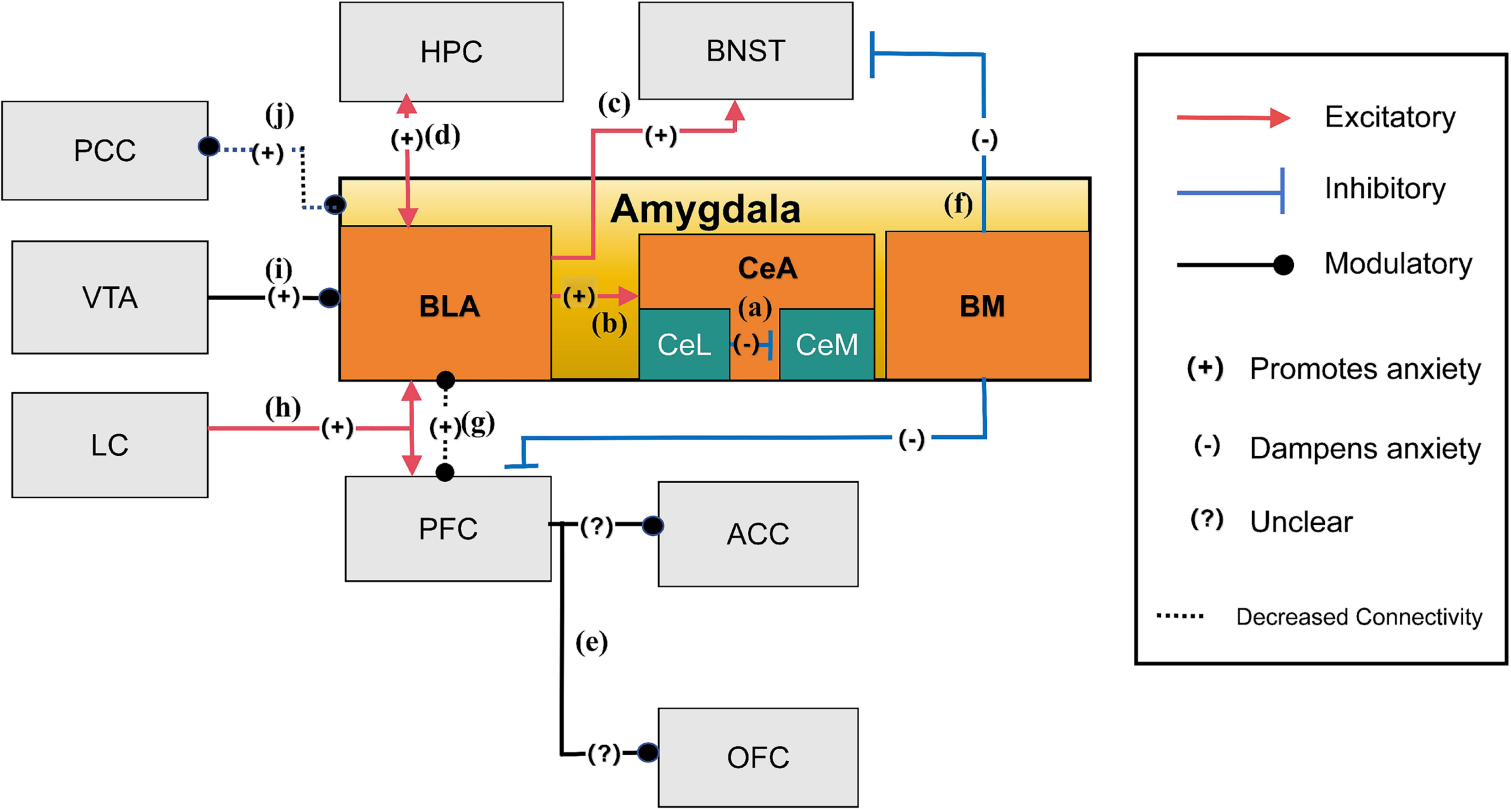
The anxiety network of amygdala (Steimer, 2002; Tye et al., 2011; Poulos et al., 2010; Calhoon and Tye, 2015; Felix-Ortiz et al., 2013; Felix-Ortiz et al., 2016; Yang and Wang, 2017; Morel et al., 2022; Babaev et al., 2018; Hahn et al., 2011). **(a)** Suppressive neurons in CeL may inhibit the activation of anxiety-promoting neurons in CeM, reducing anxiety; **(b)** The amygdala, especially the CeA, is crucial for managing anxiety. During anxiety, the BLA processes sensory information from different brain areas and relays it to the CeA; **(c)** The BLA encourages anxious states by signaling environmental cues to the BNST, leading to extended periods of anxiety; **(d)** The two-way connections between the vHPC and the BLA mainly amplify anxiety reactions by amplifying each other’s functions; **(e)** Photostimulation of the BLA projections within the mPFC has been shown to significantly increase anxiety-related behaviors. These targeted BLA fibers extend beyond the mPFC, reaching regions such as the ACC and OFC, which are also involved in the regulation of anxiety; **(f)** The BM influences anxiety by attenuating it through its connections with regions such as the adBNST and vmPFC; **(g)** The decreased functional connection between the amygdala and the PFC leads to heightened anxiety levels; **(h)** The LC is essential for anxiety and responds to stress by influencing the body’s stress response. It connects to key brain areas for anxiety, like the amygdala and PFC; **(i)** The VTA dopamine neurons project to the BLA and regulate anxiety-like behaviors; **(j)** Reduced connectivity between the amygdala and PCC leads to increased anxiety severity.

The amygdala, particularly the CeA, functions as a critical integration center where sensory information about potential threats—both conditioned (learned) and unconditioned (innate)—is evaluated, leading to the initiation of acute fear or anxiety responses. When anxiety is triggered, the basolateral amygdala (BLA) integrates both conditioned (learned) and unconditiioned (innate) sensory information from various brain regions, interprets it, and relays it to the CeA for further processing (Tye et al., 2011; Calhoon and Tye, 2015). Within the CeA, which is central to recognizing and responding to acute threat, suppressive neurons in the lateral division (CeL) may inhibit the activation of anxiety-promoting neurons in the medial division (CeM) (Figure 2a). Optogenetic studies on the BLA-CeL-CeM pathway indicated that activating the BLA terminals within the CeL (ChR2: BLA–CeA) reduced anxiety-like behaviors, as evidenced by prolonged open-arm duration in the EPMT and longer center durations in the OFT. The observed effect results from CeL neurons hindering CeM output neurons, underscoring the vital role of the CeL-CeM inhibitory pathway in limiting anxiety output (Tye et al., 2011). Within the CeA, inhibitory projection neurons are essential for conveying processed threat signals from the CeL to the CeM, which then coordinates downstream anxiety responses. Specific neuron populations, like PKCδ+ and CRF + neurons, influence anxiety-like behaviors through various mechanisms (Babaev et al., 2018; Isosaka et al., 2015; Kim et al., 2017). Additionally, components of the GABAA receptor, notably the γ2-subunit and α2-GABAARs, are also pivotal in the amygdala’s anxiety circuitry. Studies have shown that mice lacking the α2-or γ2-subunit of the GABAA receptor spend significantly less time in the light compartment of the Light–Dark Box (LDB) paradigm, highlighting the significance of these receptor systems in maintaining emotional balance (Babaev et al., 2018; Smith and Rudolph, 2012). Within the BLA, specific interneuron subtypes, such as parvalbumin (PV+), somatostatin (SOM+), and cholecystokinin (CCK+) interneurons, modulate amygdala output and anxiety-like behaviors, and facilitation of endocannabinoid signaling, respectively (Urakawa et al., 2013; Fuchs et al., 2017; Trouche et al., 2013). Moreover, glutamatergic neurons in the amygdala primarily function as excitatory elements, potentially increasing neuronal excitability in amygdala output pathways, promoting anxiety-like behaviors. However, this excitatory influence can be mitigated by the action of α7 receptors. As previous animal studies suggest, α7 receptors may downregulate these excitatory glutamatergic inputs to the amygdala, aligning with reduced anxiety-like behaviors observed in animal models treated with BNC210 (Wise et al., 2020). Furthermore, studies highlighted the role of the PKCδ+ expressing subpopulation of central amygdala neurons, which often co-express the α5 of the GABAA receptor. This subunit mediates tonic inhibition of PKCδ+ neurons in the CeA, constraining anxiety output through extrasynaptic inhibition of PKCδ+ neurons within the CeA. Notably, in states of heightened anxiety, expression of the α5 subunit decreases, leading to a loss of inhibitory regulation. Following an anxiety-inducing stimulus, the inhibitory effect mediated by the α5 receptor diminishes in PKCδ+ neurons, while it remains preserved in PKCδ− neurons. This disruption in inhibitory control is strongly correlated with increased avoidance behavior in anxiety tests, particularly ingeneralized anxiety disorder (GAD) (Botta et al., 2015; Ciocchi et al., 2010). Experiments involving selective deletion of the α5 receptor exclusively in the CeA have demonstrated a significant increase in anxiety behaviors, demonstrating that loss of α5 receptor-mediated inhibition specifically in the CeA is sufficient to increase anxiety-like behaviors. When comparing different neuron types, changes in PKCδ+ neurons show a stronger correlation with anxiety than those in PKCδ− neurons, highlighting the critical role of α5 receptors in PKCδ+ neurons for anxiety regulation. This suggests that modifications in the α5 receptors in PKCδ+ neurons may have significant implications for anxiety levels (Botta et al., 2015).

The BLA is vital in detecting environmental signals—whether conditioned (e.g., a tone paired with shock) or unconditioned (e.g., sudden loud noise)— and relaying them to the CeA and BNST. While the CeA is responsible for orchestrating immediate defensive reactions and acute anxiety initiation, the BNST mediates the sustained behavioral and physiological components of anxiety in response to ambiguous or prolonged threat, highlighting the CeA’s role in acute, stimulus-specific responses and the BNST’s role in sustained, context-dependent anxiety (Poulos et al., 2010; Calhoon and Tye, 2015) (Figures 2b,c). Neural projections from the BLA to the vHPC directly influence the expression of anxiety-like behaviors in response to anxiogenic stimulus, as demonstrated by the EPMT and OFT (Tejeda and O’Donnell, 2014; Felix-Ortiz et al., 2013; Calhoon and Tye, 2015; Figure 2d). More precisely, neural pathways from the BLA to the mPFC, extending into areas such as the ACC and orbitofrontal cortex (OFC) (Felix-Ortiz et al., 2016; Albrechet-Souza et al., 2009), are pivotal in modulating the behavioral expression of anxiety, as stimulation of these pathways can increase avoidance (Figure 2e). Moreover, the dynamic interplay between the prefrontal cortex and the CeA illustrates how a decrease in functional connectivity can reduce inhibitory control within the CeA, thereby exacerbating anxiety (Tovote et al., 2015; Birn et al., 2014). Additionally, the basomedial nucleus (BM) of the amygdala, located in the ventromedial region near the BLA, has distinct roles in modulating anxiety (Yang and Wang, 2017). It influences anxiety through connections with the adBNST and vmPFC. Notably, light stimulation of the BM has been shown to alleviate heightened anxiety, while its inhibition via optogenetic or pharmacological methods leads to increased anxiety responses (Yang and Wang, 2017; Figure 2f). In response to stress, the amygdala plays a central role in threat perception and anxiety regulation by modulating its interactions with the prefrontal cortex (PFC). Decreased functional connectivity between the amygdala and PFC has been inversely correlated with innate anxiety levels, with different PFC regions either promoting or suppressing anxiety through their bidirectional regulation with the amygdala anxiety through their bidirectional regulation with the amygdala (Calhoon and Tye, 2015; Steimer, 2002; Figure 2g). The locus coeruleus (LC) also contributes to this regulatory network by projecting into both the amygdala and PFC, increasing amygdala neuronal excitability to threat cues and influencing PFC-dependent regulation of anxiety responses. This interaction integrates the LC into the broader control system governing the amygdala and PFC’s response to stress and anxiety (Steimer, 2002; Figure 2h). Furthermore, dopamine neurons projecting from the VTA to the BLA modulate anxiety-like behaviors, as altering their activity changes exploration in anxiogenic environments. Alterations in their function provide potential therapeutic targets for anxiety and related conditions (Morel et al., 2022; Figure 2i). In individuals with anxiety disorders, alterations in connectivity patterns, particularly reduced amygdala engagement with cortical regions like the posterior cingulate cortex (PCC), correlate with clinical symptom severity scores in anxiety disorders, suggesting a distinct neurobiological underpinning for anxiety symptoms (Hahn et al., 2011; Figure 2j). This complex network of brain circuits and pathways underscores the intricate nature of anxiety responses, emphasizing the delicate balance across diverse brain regions in experiencing, responding to, and regulating anxiety.

BLA (Basolateral Amygdala), CeA (Central Amygdala), CeL (Central Amygdala Lateral part), CeM (Central Amygdala Medial part), BM (Basomedial Nucleus), VTA (Ventral Tegmental Area), LC (Locus Coeruleus), PFC (Prefrontal Cortex), PCC (Posterior Cingulate Cortex), ACC (Anterior Cingulate Cortex), OFC (Orbitofrontal Cortex), HPC (Hippocampus), and BNST (Bed Nucleus of the Stria Terminalis).

## 4 The role of LHb in anxiety regulation

The LHb plays a pivotal role in the pathophysiology of stress-related anxiety conditions (Gill et al., 2013). This bilateral epithalamic structure, situated between the dorsomedial thalamus and the third ventricle, encodes negative valence and aversive signals, contributing to the expression of anxiety-like behaviors (Hikosaka, 2010). Excessive LHb activity, often triggered by stress-induced anxiety, contributes to helplessness, loss of sensation, and reduced motivation. Stressors and adverse emotional stimuli, like social defeat and other chronic stressors frequently activate the LHb, promoting behaviors such as social avoidance and reduced exploration in novel and anxiogenic environments. The LHb receives afferent signals from key limbic and basal structures in the forebrain, including the hypothalamus, BNST, LC, and amygdala (Purvis et al., 2018; Gill et al., 2013). These inputs relay stress, reward, and emotional information, allowing the LHb to modulate behavioral responses. The LHb, in return, influences monoaminergic systems primarily via projections to the rostromedial tegmental nucleus (RMTg), indirectly impacting the VTA and dorsal raphe nucleus (DRN) (Jhou et al., 2009; Dolzani et al., 2016). These interactions regulate neural activities, influencing an individual’s susceptibility to developing anxiety-like behaviors following stress. Thus, the LHb plays a vital role in sustaining emotional equilibrium and behavioral reactions to environmental stressors related to anxiety (Gill et al., 2013).

In the neurobiological context of anxiety, the LHb interacts with diverse neurotransmitters, modulating anxiety-related behaviors. Conversely, the LHb influences neural activation and glutamate release via CRF1R and CRF2R receptors. To be specific, the CRF1R-PKA signaling pathway reduces GABAergic inhibition, leading to increased excitability and hyper-glutamate state in LHb neurons (Zuo et al., 2021; Authement et al., 2018). Intriguingly, CRF1R and CRF2R exert opposing effects on glutamatergic transmission (Zuo et al., 2021), with CRF activating the CRF2R-PKC pathway to decrease glutamate transmission in the LHb (Zuo et al., 2021). Prolonged alcohol exposure disturbs this balance by activating CRF1R and inhibiting CRF2R at the glutamatergic terminals on LHb neurons, increasing measures of anxiety-like behavior and alcohol consumption (Zuo et al., 2021). Early research suggests that modulating LHb functionality by altering glutamate or GABA transmission can induce anxiety-like behaviors. In experimental settings, administering either the GABA-A receptor activator Musc or the glutamatergic AMPA receptor blocker CNQX into the LHb prevented rats from exploring the center of an open field in an unfamiliar environment, reinforcing the link between glutamate-GABA imbalance and anxiety-related behaviors (Lecourtier et al., 2023). Additionally, suppressing LHb activity has been shown to increase open arm exploration in the elevated plus maze in stressful situations, suggesting its potential as a therapeutic target (Gill et al., 2013). For instance, yohimbine-induced increases in NE and CRH release in the BNST and amygdala increases neuronal firing in the LHb, correlating with the expression of anxiety-like behaviors. This discovery presents a novel pharmacological approach for targeting the yohimbine mechanism in developing new anxiety treatments focused on the LHb (Gill et al., 2013).

**Figure 3:**
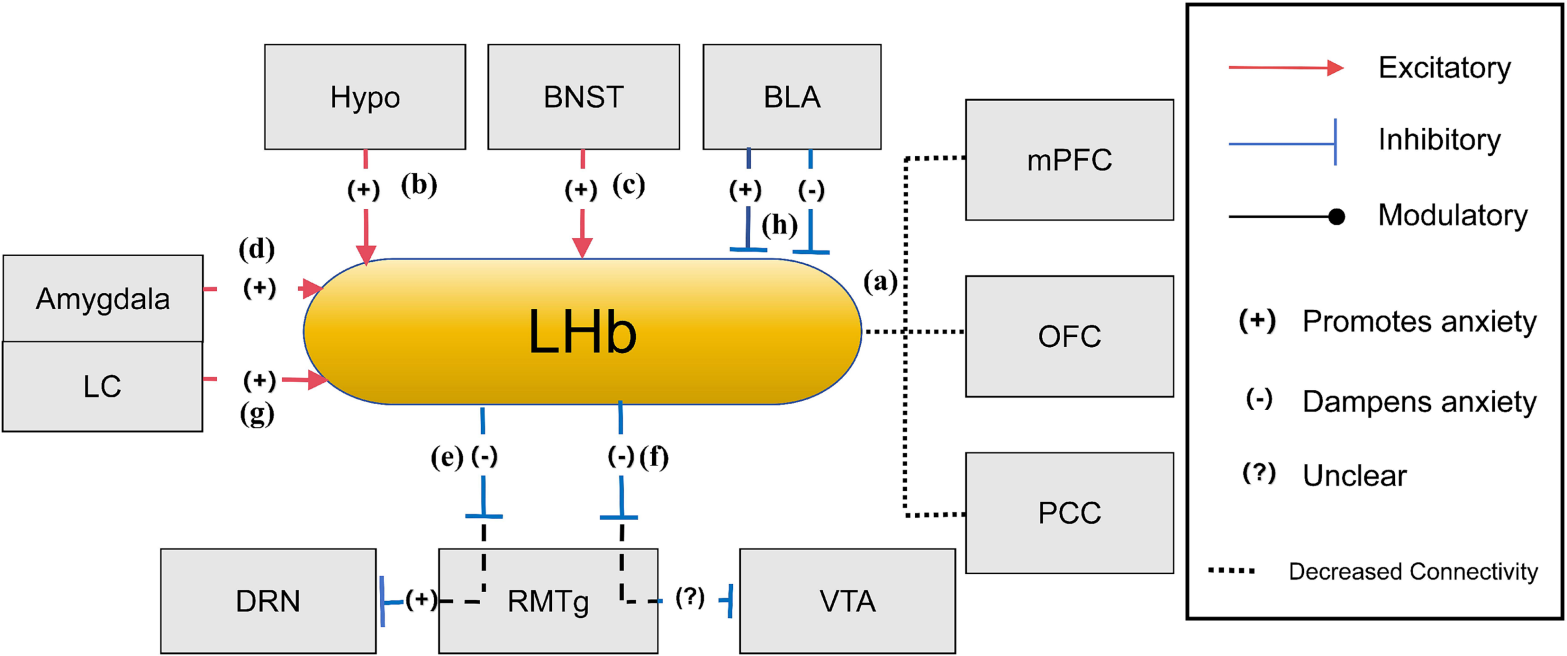
The anxiety network of LHb (Ma et al., 2020; Gill et al., 2013; Dolzani et al., 2016; Purvis et al., 2018; Yang et al., 2021). **(a)** When experiencing anxiety, the diminished connection between the LHb and the PFC, OFC, and cingulate cortex hinders threat assessment and reward processing, and a lesser link with the OFC, VTA, and PCC results in less effective information processing and focus; **(b)** During states of anxiety, increased activity of the hypothalamus, particularly the lateral region, may lead to enhanced stimulation of the LHb, which is a pivotal stress response pathway; **(c)** The BNST in the stria terminalis amplifies LHb activity through the secretion of neurotransmitters linked to stress, thereby amplifying LHb’s function in managing stress and anxiety; **(d)** The amygdala influences LHb activity through stress signal transmission, thereby playing a role in the LHb’s function of managing responses to fear and anxiety; **(e)** When the LHb is active, it transmits inhibitory signals to the DRN through the RMTg, this action leads to decreased serotonin output from the DRN, which is typically associated with enhancing anxiety due to the reduced mood stabilization serotonin provides; **(f)** The LHb exerts an inhibitory influence on the RMTg, leading to reduced RMTg activity. With the RMTg’s inhibitory control over the VTA being weakened, there is an indirect increase in dopamine release from the VTA. This modulation can potentially alleviate anxiety or could contribute to the development or exacerbation of anxiety, as the brain’s reward system can become dysregulated; **(g)** The LC plays a critical role in anxiety-related behaviors through its noradrenergic pathways to the LHb, with decreasing NE release in the LHb being essential for sustaining these behavioral states; **(h)** LHb regulates BLA activity through projections to midbrain dopaminergic neurons. Presynaptic GABAB receptor inhibition in LHb increases DA and 5-HT release in the BLA, promoting anxiolytic effects, while postsynaptic GABAB receptor inhibition decreases DA and 5-HT release, leading to anxiogenic effects.

LHb exhibits an intricate web of neural connections, particularly during anxiety states. Neuroimaging research reveals stronger connections between the habenula and frontal cortex areas, including the orbitofrontal cortex (OFC) and cingulate cortex, both critical for threat evaluation and reward processing. These connections are often altered in individuals with anxiety disorders, potentially contributing to difficulties in threat evaluation and attentional control. Conversely, reduced connectivity between the LHb and regions such as the OFC, VTA, and posterior cingulate cortex (PCC) could impair information processing and attentiveness, hallmark features of anxiety states (Ma et al., 2020; Figure 3a). LHb receives afferent input from key limbic structures, including the hypothalamus, BNST, and amygdala (Gill et al., 2013). Heightened LHb activity during anxiety might result from excessive stimulation of core afferent cells, particularly in the lateral hypothalamus, a key region in stress responses (Gill et al., 2013; Figure 3b). Additionally, secondary afferent pathways involving the extended amygdala and BNST contribute to excessive LHb excitation during stress (Lawther et al., 2020; Figures 3c,d). The LHb exerts significant influence in the dopaminergic system via its connections with the VTA (Jhou et al., 2009). Its primary glutamatergic projections travel to the tail of the VTA (tVTA) via the fasciculus retroflexus (FR). LHb activation stimulates GABAergic neurons within the tVTA, reducing tVTA output, which in turn relieves the inhibitory pressure on dopamine neurons, ultimately enhancing dopaminergic activity (Jhou et al., 2009; Gill et al., 2013). The tVTA, also known as the RMTg, serve as the LHb’s primary output pathway, regulating both dopaminergic and serotonergic neurons within the VTA and the dorsal raphe nucleus (DRN) (Dolzani et al., 2016). Experimental studies using the inescapable tailshock (IS) paradigm highlight the LHb-RMTg pathway’s role in stress response and hypothalamic–pituitary–adrenal (HPA) axis activation (Dolzani et al., 2016). This pathway mitigates the adverse effects of stress by inhibiting dopaminergic neurons and serotonergic neurons, both of which modulate mood, motivation, and responses to threat, processes central to anxiety (Jhou et al., 2009; Dolzani et al., 2016; Figures 3e,f). Furthermore, research also underscores LC as a crucial modulator of stress and anxiety. LC projections to the LHb regulate norepinephrine (NE) levels, directly influencing anxiety-related behaviors. Increased NE activity in the LHb is associated with heightened arousal and anxiety, as demonstrated by acoustic startle response (ASR) tests (Purvis et al., 2018; Figure 3g). The LHb also influences BLA activities via its interactions with midbrain dopaminergic neurons. Within this circuitry, presynaptic GABAB receptors activation in the LHb inhibits neurotransmitter release by obstructing calcium channels. Pharmacological inhibition of these presynaptic receptors, such as those with CGP36216, reduces the activity of LHb neurons, resulting in increased dopamine (DA) and serotonin (5-HT) in the BLA, producing anxiolytic effects by observing increased open-arm time and increased percentage of open-arm entries (Yang et al., 2021). Conversely, the activation of postsynaptic GABAB receptors reduces LHb excitability through GIRK channels. Inhibiting these postsynaptic receptors (such as through CGP35348) enhances LHb activity, reduces DA and 5-HT release in the BLA, and produces anxiety-like behaviors by observing decreased open-arm time and increased percentage of open-arm entries. The circuit emphasizes the role of the LHb in modulating emotional responses mediated by the BLA (Yang et al., 2021; Figure 3h).

Hypo (Hypothalamus), BNST (Bed Nucleus of the Stria Terminalis), VTA (Ventral Tegmental Area), LC (Locus Coeruleus), PFC (Prefrontal Cortex), PCC (Posterior Cingulate Cortex), OFC (Orbitofrontal Cortex), DRN (dorsal raphe nucleus), RMTg (Rostromedial Tegmental Nucleus), LHb (Lateral Habenula).

## 5 The role of other regions in anxiety regulation

### 5.1 Hippocampus

In addition to key areas such as the amygdala, BNST, and LHb, numerous other brain regions or pathways are intricately involved in the neural circuitry underlying anxiety-like behaviors. A prominent structure is the HPC, which, in conjunction with various brain areas, encodes contextual information about potential threats and modulates anxiety-like behaviors based on past experiences and spatial context. The HPC encodes contextual information about potential threats, a fundamental function in the brain’s evaluation of uncertain and future-oriented dangers. The dorsal HPC (dHPC) is primarily associated with cognitive abilities like learning and memory, while the ventral HPC (vHPC) is more involved in regulating emotions, illustrating its distinct contribution to context-dependent aspects of anxiety (Fanselow and Dong, 2010). Structurally, the HPC is subdivided into three key sections: CA1, CA3, and the dentate gyrus, each serving unique roles in learning and memory. Notably, the DG and CA3 have been closely linked to anxiety-related behaviors (Engin et al., 2016). By encoding contextual information from past experiences, the HPC influences the perception of potential threats. This information is transmitted to the prelimbic cortex (PL) in the PFC, often through synchronized neural activity, particularly theta waves, which synchronize during exposure to anxiogenic contexts and correlate with anxiety-like behavioral states in both rodents and humans. Research indicates the coordinated function between the vHPC and PFC during anxiety-inducing situations. The septohippocampal axis, which heavily involves the HPC, is another crucial system in stress-induced anxiety, balancing opposing anxiolytic and anxiogenic processes. Neural projections from the vHPC to the lateral septum (LS) transmit anxiety-related signals, leading to the suppression of neuronal activity in the hypothalamus, especially in the paraventricular nucleus and the periaqueductal gray area. This suppression reduces prolonged avoidance of open arms in the EPM, a key behavioral marker of anxiety, as demonstrated by pharmacological inhibition of vHPC or LS activity with muscimol (Kim et al., 2013; Calhoon and Tye, 2015; Tovote et al., 2015). And when reduced, it amplifies persistent anxiety while strengthening vHPC-mPFC connectivity, further implicating the vHPC as a key driver of anxiety-related processes in the mPFC (Calhoon and Tye, 2015; Herry et al., 2008). In addition, research has demonstrated that activation of the BLA-vHPC pathway produces anxiety-like behaviors, while inhibition of this pathway alleviates them. This demonstrates that activity along the BLA-vHPC axis bidirectionally controls social interaction time, a behavior often disrupted in anxiety (Felix-Ortiz and Tye, 2014). Lastly, neural projections from the ventral CA1 (vCA1) region of the HPC to the lateral hypothalamic area (LHA) rapidly modulate avoidance behavior in anxiogenic environments through glutamate-producing neurons. The discovery of this unique vCA1-LHA pathway, which swiftly affects anxiety behaviors, offers promising avenues for new approaches to treating mood and anxiety disorders (Jimenez et al., 2018).

### 5.2 Pfc

PFC also plays a crucial role in anxiety regulation (Calhoon and Tye, 2015). Studies using optogenetics have demonstrated that the mPFC-BLA pathway functions bidirectionally. The activation of this pathway escalates anxiety symptoms and reduces social engagement, while its suppression produces the opposite effect. This implies that the mPFC plays an adaptive role in anxiety regulation and social activities by dynamically modulating BLA activity based on environmental contexts. For instance, in the presence of real threats, the mPFC might enhance BLA activity to increase anxiety and vigilance, whereas in safe environments, it can reduce BLA activity to reduce anxiety and promote social engagement (Felix-Ortiz et al., 2016). Furthermore, recent findings have also identified a neural circuit from the PFC to the CEA in both primates and humans that contributes to increased anxiety. This anxiety-promoting effect is partly attributed to an uninhibited CEA, which results from weakened functional connections between prefrontal areas and the CEA (Birn et al., 2014). Increased activity in the right PFC correlates with adverse emotions and anxiety, emphasizing the PFC’s essential part in regulating anxiety and stress responses. Notably, this highlights the importance of brain lateralization within PFC mechanisms, particularly in human models (Steimer, 2002; Balderston et al., 2020).

### 5.3 LHA

The lateral hypothalamic area (LHA) is also a crucial brain region involved in anxiety modulation. Neurons within the LHA, particularly those responsible for melanin-concentrating hormone (MCH) secretion, has been strongly associated with anxiety-like behaviors (He et al., 2022). MCH, a peptide primarily located in the LHA, is associated with anxiety-like behaviors. Chronic stress increases MCH expression, which in turn exacerbates anxiety-like symptoms (Jang et al., 2018). Studies indicate that activating MCH neurons in the LHA distinctly intensifies anxiety responses, highlighting the role of the LHA-BLA pathway in anxiety regulation (He et al., 2022). Additionally, the LS and its GABAergic projections to the LHA are also crucial for anxiety regulation. Anxiety heightens the activities of GABAergic neurons within the LS, and optogenetic or chemogenetic inhibition of these neurons reduces anxiety-like behaviors in mice. Conversely, the chemogenetic stimulation of LH-projecting LS neurons induces anxiety-like behaviors in previously untrained mice. Fiber photometry recordings further reveal that LS neurons projecting to the LHA respond dynamically to anxiety-related stimuli, such as von Frey tests, EPMT, or OFT, thereby influencing anxiety-related actions in animals (Wang et al., 2023).

## 6 Discussion

**Figure 4:**
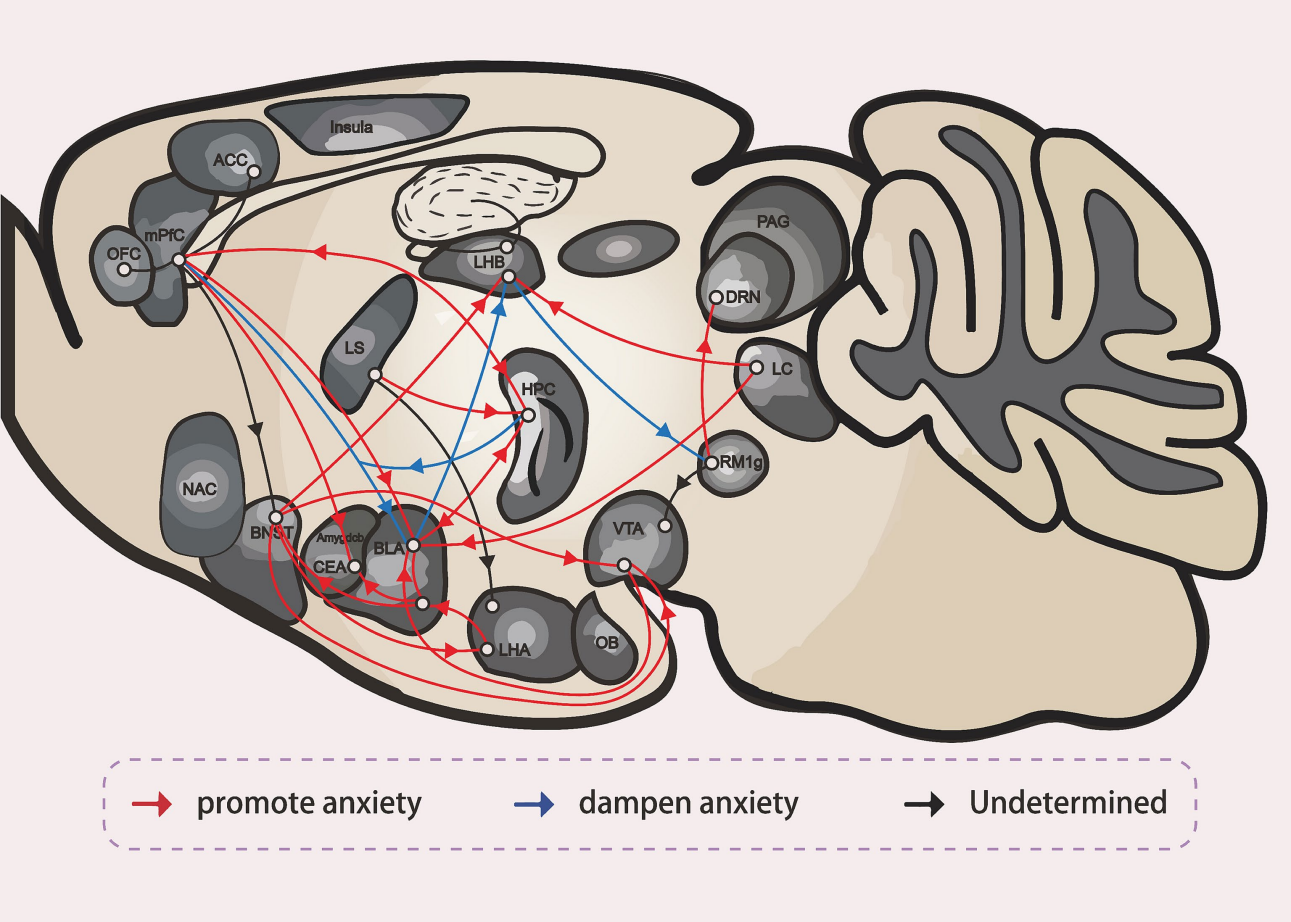
The neural circuit of anxiety (Hu, 2016).

Anxiety disorders are among the most common neurological conditions today, posing significant challenges for both diagnosis and intervention. However, recent advances in neural circuit research have greatly enhanced our understanding of these disorders and opened new avenues for treatment. This review provides a concise overview of major anxiety-related brain regions, including the BNST, amygdala, LHb, HPC and others, and their connectivity, highlighting their roles in the neurobiological mechanisms underlying anxiety (Figure 4). Additionally, this review discusses the roles of a variety of interneurons, local receptors, neurotransmitters, and divergent neural pathways in these brain regions, all of which contribute to the emergence and regulation of anxiety-like behaviors. These make significant insights into the complexity of anxiety pathology and offer multiple promising targets for therapeutic intervention and drug development. By integrating key findings from recent studies, this review not only deepens our understanding of anxiety-related circuitry but also points to new directions for research and clinical translation. Ultimately, this work bridges novel neurobiological discoveries with future strategies for the diagnosis and treatment of anxiety disorders, making a meaningful contribution to the field.

HPC (Hippocampus), mPFC (medial Prefrontal Cortex), BLA (Basolateral Amygdala), CeA (Central Amygdala), BNST (the Bed Nucleus of the Stria Terminalis), ovBNST (oval nucleus of the Bed Nucleus of the Stria Terminalis), vlBNST (ventrolateral nucleus of the Bed Nucleus of the Stria Terminalis), LHA (the Lateral Hypothalamus), VTA (the Ventral Tegmental Area), ACC (Anterior Cingulate Cortex), OFC (Orbitofrontal Cortex), RMTg (Rostromedial Tegmental Nucleus), DRN (dorsal raphe nucleus), LC (Locus Coeruleus), LHb (Lateral Habenula), LS (Lateral Septum), NAc (Nucleus Accumbens), PAG (Periaqueductal Gray), OB (Olfactory Bulb).

In addition to the commonly studied brain regions, several other areas implicated in anxiety are under investigation, though their neuroregulatory mechanisms remain unclear and need further exploration. Notably, the insula cortex, particularly its anterior region, has been implicated in anxiety, as it plays a crucial role in anticipating negative events and evaluating risk. Recent studies in rodents, though limited in scope, have begun to investigate its roles in rodents (Klein et al., 2021). The paraventricular nucleus (PVN) of the hypothalamus serves as a central hub in coordinating stress responses and is a key component of the LHPA’s “stress cycle,” which is closely linked to the serotonergic signaling. The PVN is essential for regulating stress-related physiological functions, mood, and cognition, all of which are critical in the context of anxiety disorders (López et al., 1999). Moreover, there exists an inverse relationship between the integrity of the uncinate fasciculus (UF) and characteristic anxiety (Baur et al., 2012). The olfactory bulb, with projections to the amygdala and certain GABAergic neurons have been linked to anxiety disorders and depressive states, possibly clarifying the anxiety-reducing effects (Monti and Liebowitz, 2022). The median raphe nucleus (MRN) is vitally important in managing anxiety, particularly through its serotonergic projections to dHPC, which influence anxiety-like behaviors. Additionally, the MRN also communicates with other anxiety-related brain regions, including the amygdala complex, indicating its broad involvement in anxiety regulation (Abela et al., 2020). These findings emphasize the complex interplay among multiple brain areas and neural circuits involved in the modulation of anxiety. They also highlight the urgent need for expanded research to uncover the mechanisms underlying these interactions. Understanding the brain’s anxiety-regulatory networks offers significant promise for informing clinical strategies aimed at symptom management and improving treatment outcomes. Each neural circuit and its projection play distinct roles in either promoting or inhibiting anxiety-like behaviors. To effectively modulate these influences, it is essential to understand the nuanced mechanisms of connectivity and interaction across these regions.

While the identification of anxiety-related brain regions and circuits is foundational, translating these discoveries into clinical applications requires bridging the gap between neurobiological insights and therapeutic innovation. Recently, different emerging new technologies have begun to address these challenges, offering novel methods to study neural circuits. In such contexts, techniques like two-photon microscopy, functional magnetic resonance imaging (fMRI), and neurofiber technologies like multi-electrode arrays have greatly contributed to an expanding understanding of neural circuitry, especially in light of synaptic plasticity and network dynamics. Two-photon microscopy enables dynamic observation of synaptic changes during learning and memory, illustrating how synaptic connections are strengthened or weakened. While learning is associated with the formation and stabilization of new synapses, forgetting involves the weakening or even elimination of these connections. Understanding such cellular mechanisms is crucial for designing therapeutics that will protect or restore cognitive function in neurodegenerative diseases, such as Alzheimer’s disease (Xiong et al., 2023). In contrast, two-photon microscopy is limited by its shallow penetration depth and temporal resolution, is largely restricted to animal models. fMRI maps large-scale neural circuits and detects significant alterations brain connectivity associated with major psychiatric disorders, such as anxiety and depression. This has generated an interest in therapeutic interventions, for example, transcranial magnetic stimulation (TMS) and neurofeedback. Nonetheless, fMRI is hindered by poor temporal resolution and its indirect measurement of neural activity, which limits its ability to capture real-time neuronal dynamics (Mizutani-Tiebel et al., 2022). Multi-electrode arrays offer high temporal and spatial resolution, enabling detailed investigation of coordinated neuronal activity and the development of real-time modulation therapies. However, their invasive nature, challenges in achieving chronic stability, and the complexity of data analysis constrain their broader application (Lee et al., 2023). Despite these individual limitations, these technologies are complementary. As they continue to evolve, they promise to deepen the understanding of neural circuits and their roles in both health and disease. These innovations offer new insights into psychological mechanisms that underlie psychiatric disorders and toward more effective treatments.

The integration of cross-species research is critical to validate these neurotechnological advancements and ensure their relevance to human anxiety disorders. The translation of findings in neural circuit research from mice to primates and humans raises enormous hurdles. While mouse models remain indispensable for foundational studies, they are inherently limited in their ability to capture the full complexity of human psychopathological processes, particularly those engaged during cognitive-behavioral therapy (CBT) interventions (Robinson et al., 2019). For instance, while rodent elevated plus maze tests (EPMT) approximate human avoidance behaviors, they lack the cognitive complexity of human anxiety manifestations like rumination, which engages dorsolateral prefrontal circuits (Yuan et al., 2018). Moreover, the functional homology of cortical areas across species is limited, complicating the direct translation of neural findings from rodents to humans. To improve translational validity, it is essential to explore the extent to which anxiety-related traits in mice correspond to human anxiety conditions. Notably, a conserved neural mechanism exists across species in the bidirectional connectivity between the BNST and the amygdala in both mice and humans. The circuit forms a bidirectional route for signal transmission, establishing a complex regulatory system. Neural analysis reveals neural pathways from the BNST sub-nuclei to the amygdala nuclei, and the reverse, establishing the link. Emerging technologies like iPSC-derived neuronal cultures are now bridging these gaps (Sfakianou, 2019). In both species, distinct amygdala nuclei (CeA, BLA) exhibit comparable regulatory impacts on BNST activity. The amygdala plays a central role in modulating anxiety. As an illustration, the GABAergic projection from the CeA diminishes the inhibitory effect of BNSTALG, whereas the excitatory input from BLA enhances it (Van de Poll et al., 2023). The cross-species resemblance in anxiety-regulation pathways highlights an evolutionary conservation of mechanisms, making mouse models valuable for studying human anxiety (Van de Poll et al., 2023). Besides, experimental paradigms designed for mice c often have translational relevance. Techniques such as telemetry can capture physiological parameters in mice that reflect human anxiety responses under threat (Hohoff, 2009). Mice are also amenable to MRI, enhancing their utility in translational research. Moreover, within behavioral frameworks, unconditioned reactions (such as the elevated plus/zero maze, light - dark box, open field, anxiety - defense test battery, and predator tests) similarly contribute to the imitation of human anxiety-related actions (Hohoff, 2009). A comprehensive comparative analysis of the neural pathways, neurotransmitter pathways, and behavioral responses in both species is needed to fully understand their interplay within the anxiety spectrum. While rodent models provide valuable insight, non-human primates offer a closer approximation to human neuroanatomy and cognitive functions. Their complex social behaviors and decision-making processes afford deeper understanding of higher-order mental functions. However, the differences in brain structures and functional areas may limit the generalizability of primate findings to humans. Moreover, the high costs and ethical concerns associated with primate research restrict its broader application (Quigley, 2007; Nelson and Winslow, 2009). To bridge these gaps, researchers are leveraging advanced technologies such as optogenetics and fMRI in both rodent and primate studies. These technologies allow for comprehensive comparison of neural circuit functions across species, helping to the identification of common mechanisms (Klink et al., 2021). Additionally, standardized experimental designs and analytical methods across humans and animal studies improve the evaluation of potential therapies. For example, simultaneous drug screenings in human volunteers and primates can more accurately assess efficacy and side effects. By combining these advanced technologies with cross-species research methods, scientists are progressively overcoming the translational barriers in neural circuit research, paving the way for more effective therapeutic interventions.

These multidisciplinary efforts—spanning circuit dissection, technological innovation, and cross-species validation—culminate in the development of pharmacological strategies that directly target anxiety-related neural mechanisms. Pharmacological strategies primarily target neurotransmitter systems implicated in anxiety-related circuits. In animal models, such as the mouse, benzodiazepines act on the GABA-A receptor to increase chloride channel opening the rate of opening of the chloride channel, resulting in excessive hyperpolarization of the postsynaptic neuron. This reduces neuronal excitability and leads to decreased anxiety-like behaviors in paradigms such as the elevated plus-maze and light–dark box tests. In human, dysregulated GABAergic signaling is implicated in pathological anxiety states, and benzodiazepines restore inhibitory tone by amplifying endogenous GABA effects (Rudolf et al., 1999). Benzodiazepines amplify GABA’s impact, offering rapid anxiety relief by modulating inhibitory neurotransmitters (Pallanti et al., 2024). The serotonergic system is another key therapeutic target. Selective serotonin reuptake inhibitors (SSRIs) elevate serotonin levels in the synaptic cleft by blocking the serotonin in mice, contributing to alleviate anxiety symptoms via the serotonin system and making mice show more exploration in open-field tests. The serotonergic system also impacts mood in human by SSRIs, showing the bridge between mouse models and human effects (Hicks et al., 2015). Notably, recent advances have focused on subtype-specific serotonin receptor modulation. The 5-HT1A receptor exists as both autoreceptors on serotonergic neurons in the raphe nucleus and heteroreceptors on non-serotonin-containing neurons in forebrain regions such as the septum, hippocampus, or cortex. Activation of these two types of receptor typically induces membrane hyperpolarization via G protein-gated inwardly rectifying potassium channels, reducing neuronal excitability. This is particularly significant in the dorsal raphe nucleus, a key site for serotonergic regulations (Gross et al., 2002; Hoyer, 1988). By influencing neuronal firing in limbic and septal regions, 5-HT1A agonists can modulate both nxiogenic processes and hypothalamic neurotransmission relevant to anxiety regulation (Ohno, 2010). Many promising anxiolytic drugs in this category, such as buspirone, ipsapirone, gepirone, and SM-3997 (Feighner and Boyer, 1989), demonstrate high selectivity for these 5-HT1A receptor subtypes and show strong potential for clinical application. Specifically, the anxiolytic effects of 5-HT1A-targeting drugs may depend not only on their agonist properties but also on potential antagonistic actions (Feighner and Boyer, 1989). A growing body of research conceptualizes anxiety disorders as dysregulations in managing inappropriate anxiety responses, shifting the focus of translational neuroscience toward understanding mechanisms of anxiety regulation. This shift has led to the development of novel therapeutic agents, including selective 5-HT1A receptor partial agonists, which hold significant potential in the treatment of generalized anxiety disorder (GAD) (Ren et al., 2024). Beyond monoaminergic systems, glutamatergic modulation has emerged as a promising frontier. Drugs targeting the glutamatergic system, particularly NMDA receptors, have also shown promise. Ketamine, a non-competitive NMDA receptors antagonist, has gained attention for its rapid and robust anxiolytic effects in reducing treatment-resistant anxiety. In animal studies, ketamine reduces anxiety-like behavior like marble-burying tests, and has shown similar efficacy in in humans (Ray and Kious, 2016).

While preclinical research has laid a robust foundation for understanding anxiety neurobiology, translating these insights to clinical practice demands integration of human neuroimaging, genetics, and clinical evidence. Human neuroimaging studies, such as functional magnetic resonance imaging (fMRI), have revealed that individuals with anxiety disorders exhibit hyperactivation of the amygdala and bed nucleus of the stria terminalis (BNST) during threat processing, accompanied by reduced functional connectivity with the prefrontal cortex (PFC), particularly its dorsolateral and anterior cingulate subregions (Figel et al., 2019; Wang et al., 2021). These aberrant connectivity patterns correlate with symptom severity, positioning them as potential neuroimaging biomarkers for diagnostic and therapeutic monitoring. Genetic investigations have further identified conserved risk variants in genes like BDNF and GABRA2, which modulate amygdala reactivity and stress resilience across species (Merikangas et al., 2022; Loewenstern et al., 2019), underscoring the evolutionary conservation of anxiety-related neural mechanisms. Clinically, neurostimulation therapies have emerged as promising interventions. Transcranial magnetic stimulation (TMS), which enhances PFC inhibitory control over the amygdala, has demonstrated efficacy in reducing anxiety symptoms in clinical trials (Gay et al., 2022). Meanwhile, deep brain stimulation (DBS) of the BNST is being explored to normalize hyperactive anxiety circuits in treatment-resistant cases (Dijk et al., 2013).

However, translating preclinical findings faces unique challenges due to the complexity of human cognitive processes, such as rumination, which engage cortical circuits underrepresented in rodent models (Wong et al., 2023). Non-human primate studies and induced pluripotent stem cell (iPSC)-derived neuronal cultures are helping bridge this gap by validating conserved mechanisms, such as bidirectional BNST-amygdala connectivity (Emborg et al., 2013). Looking forward, advancements in multi-omics and AI-driven neuroimaging analysis hold promise for developing personalized, circuit-based therapies that integrate genetic, neural, and behavioral data to optimize anxiety treatment strategies (Ali, 2023).

Finally, investigating the intricate neural networks that governs anxiety provides deep insights into both understanding and treating this pervasive mental health issue. By examining the complex interactions among different brain areas and their neuroregulatory processes, researchers are working to find out targeted mechanisms for anxiety-like behaviors. By the use of cutting-edge technologies in neural circuit research, the use of diverse experimental models, and the development of novel pharmacological interventions, clinicians are increasingly equipped to provide personalized and effective care. This approach fosters hope and significant progress in managing anxiety, paving the way for more efficient, customized treatment strategies.
